# Case of Chronic Hydrocephalus, in Which Recourse Was Had to Tapping, for the Purpose of Evacuating the Fluid

**Published:** 1824-12

**Authors:** William Money

**Affiliations:** Surgeon to the Royal Metropolitan Infirmary for Children, to the Asylum for the Recovery of Health, &c.


ART. III.—*Case of Chronic Hydrocephalus, in which Recourse was had to Tapping, for the purpose of evacuating the Fluid.* By WILLIAM MONEY, Esq. Surgeon to the Royal Metropolitan Infirmary for Children, to the Asylum for the Recovery of Health, &c.

THE accompanying case, although it does not present any features of absolute novelty, nevertheless may prove interesting to your readers, as one of the few recorded in which the brain has been tapped. You will perceive that, in this instance, the head was punctured eleven times within ten weeks, and nearly forty ounces of fluid evacuated. Without speculating on the chances of this method being ever so far improved as to admit of general adoption in these unfortunate cases, I may be permitted to remark, that I regard it as a duty to place the one which has occurred to me on record; as it is only by the accumulation of facts that we are ever likely to come to any satisfactory conclusions.

October 15, 1822.—James Richard Hunt, born on the 25th December, 1821. At birth, his head was considered to be larger than natural, but in other respects he was well-formed and healthy. The head gradually augmented in size, his health became deranged, and recourse was had to medical advice; but his calamities continued to increase. In August last, he was admitted, under Dr. Webster's care, at the Metropolitan Infirmary for Children; and, by treatment pursued by him, the health was perfectly restored, but the head continued to get larger, so much so, that, within the last three weeks, the circumference of it had augmented one inch. My colleague, Dr. Webster, now considered the case as hopeless, and, in a consultation with myself, we decided on taking the opinions of the other medical officers of the establishment. This was done on the 13th of October; when it was agreed that medical treatment offered no hope of relief, and accordingly it was determined to attempt a cure by reducing the bulk of the head, through the means of tapping.

The following statement is the exact size of the head, as measured on the day previous to the operation; and also a detail of other circumstances connected with the case:—The weight of the body, fourteen pounds ten ounces, including his apparel. The weight of his head, from six pounds and a half, to six pounds and ten ounces. The greatest circumference of the head, twenty-three inches. From the anterior point of the tip of one ear, carrying the thread around the occiput to the anterior point of the other ear, fourteen inches. From the anterior point of one ear, carrying the string around the forehead to the same point of the other ear, is nine inches and a half. From the posterior edge of one ear, carrying the string over the parietal bones to the same point of the other ear, is fourteen inches and a half. From the base of the nose along the vertex to the atlas, is sixteen inches. The division in the os frontis, is from two inches and a half to three inches. The division from the parietal on each side to the frontal, varies from two to three inches. The division from one parietal to the other, varies from one inch and three-quarters to two inches and a quarter. The posterior fontanel, and the division between the parietal bones and the occiput, measures much less. The forehead projects very considerably over the face; and, in consequence of the pressure upon the upper part of the orbits, the eyes are so much thrust downwards as scarcely to be seen; they have the appearance of those of a person in *articulo mortis*,—flat, semi-closed, and limpid. The face is small, and of a triangular figure. The cranium, except where above described, is as perfect and ossified as in other children of the same age. The spine is perfectly natural. The temperature of the body corresponds with that of other children; and the circulation is well carried on at the extremities. He craves for food, and is seldom satisfied. The bowels are torpid, and the urine is scanty. His respiration is regular, but inclined to be laborious. He does not rise his head, which is very hot to the touch; and his voice is faint. Fluctuation is very evident in the divisions, and, upon varying the posture of the head, the movement of fluid within the cranium is very perceptible.

At three o'clock this day, in the presence of my colleagues and several pupils, I introduced a very small, fine, flat trocar, about the size of a couching needle, in a valvular direction, into the right lateral part of the fontanel, near the sagittal suture. The instrument was introduced about an inch and a quarter, and, immediately on withdrawing the stilette, the fluid flowed out *guttatim*; afterwards, in a gradual but gentle stream; and lastly, in drops rapidly succeeding each other, but without the slightest tinge of blood, or of any other extraneous matter. Four ounces and a drachm, by measure, of a clear straw-coloured fluid were evacuated in the space of five minutes: the sides of the head all the time were gently supported by the hands of Mr. Bampfield. The child did not seem to suffer any pain or uneasiness during the operation; the pulse beat 140 in a minute, about the same as it usually did before the operation; the skin was all along naturally warm, and the breathing as usual, though the infant foamed a little at the mouth; but this the mother said it had often done before. Whilst the water was evacuating, the eyes appeared to be a little more animated; the pupils were a little more visible; and, in a general point of view, the child appeared as if it felt relieved. Two small pieces of adhesive plaster were placed cross-wise on the wound, and the whole head supported by an uniform bandage. The child was left to repose quietly, and to be kept in the horizontal posture. No medicine to be given.

Six o'clock P.M.—Very comfortable.

Ten o'clock P.M.—Sleeping, with less sonorous respiration than formerly, and has cried "loud" for food; a circumstance most gratifying to the parents, the child, of late, having "mourned" more than cried out.

16th, eight o'clock A.M.—Seems refreshed from his sleep; is more animated, and has required food only once; breathes with regularity and freedom; bowels moved once without medicine. Pulse natural, though rather quick (154); no thirst; makes water well. The head feels less hot, and the countenance is very good.

Four o'clock P.M.—Very comfortable.

Eleven o'clock P.M.—In a comfortable sleep.

17th, Meridian.—Has had two natural evacuations. Is in every respect much better, evincing increased sensibility by crying when dissatisfied, and smiling when pleased. The frontal projection is much diminished; consequently the eyes are more in view, and the pupils are continually to be seen; the face also, from the same cause, is less triangular, the cheeks wider and plumper, with a slight florid hue. The veins about the temples, upper part of the nose and orbits, are nearly empty; the head cool. The bandage remains a firm and uniform support.*

18th.—Pulse 125; has had one stool only; in other respects much as yesterday, excepting that his powers of vitality generally appear to be less than yesterday.—To have a little castor-oil in the evening, if necessary.

19th, three o'clock P.M.—He has had two evacuations without the oil; slept well and naturally; pulse 124. But there is still an apparent want of vitality about him. The bandages were removed, and the frontal and parietal bones were found to be much approximated, the integuments flaccid, and the circumference of the head measured twenty-two inches. It was determined to introduce the trocar again, which was accordingly done on the left side, nearly opposite to the first puncture; but, owing to a small spicula of bone coming in contact with the instrument, and thereby impeding its free admission, I relinquished the operation, but not until about two drachms of fluid

---

* This day I received the following letter from Mr. BRANDE:—

"My dear Sir,—Like all other encysted fluids, that which you have sent me is a weak solution of albumen, but so weak, that it scarcely acts upon the most delicate tests. Muriate of soda is very perceptible in it.

"Your's always, W. T. BRANDE."

were evacuated. A great degree of langour supervening, adhesive plaster and a firm bandage were again carefully applied. The pulse was 120, after the bandage was applied.

Night.—Has rallied, and is comfortable.

20th.—About two desert-spoonsful appear to have escaped through the opening. The bowels are freely open, and he is in every respect as reported on the 17th. Upon inquiring more minutely for the cause of the unfavourable appearances which existed during the last two days, I find that he has cut his first tooth last night or this morning; and that, since it appeared, he has changed for the better.

21st.—There are other teeth coming through, and he is evidently labouring under irritation from this cause. Bowels open. Dr. Webster, who has seen him, has prescribed for him.

22d, three o'clock P.M.—Bowels open, pulse 144, and in every respect he is better. The circumference of the head is twenty-two inches. Introduced the trocar on the right side, near the first puncture, and drew off five fluid ounces, of a deeper straw colour than before. Whilst replacing the bandage, he became so faint, cold, and exhausted, that he had the appearance of dying; but, by the use of stimulating measures, he gradually recovered.

Six o'clock P.M.—Quite recovered. The cranium is very flaccid, and the bones are distinctly separate.

23d, eight o'clock A.M.—Is much better to-day, and is more lively. Pulse 125; two sufficient evacuations. The head looks much smaller, and feels much lighter.

From the 24th to the 27th, the child remained perfectly well, and gained flesh, with florid lips and cheeks. Bowels open without medicines, and the pulse varying from 140 to 148.

28th.—Circumference twenty-two inches and a half; the head again tense, but not so much so as before the first operation. Stools natural; pulse 144; and in other respects much as usual. Introduced the trocar on the left side, between the frontal and parietal bones; and, from the obstruction which there was to the exit of the fluid, I conclude that a portion of cerebral matter must have entangled the instrument: however, at the expiration of seven minutes and a half, three fluid ounces were collected. The child then became cold, and disposed to faint, which induced me to withdraw the instrument, and close the wound.

29th.—Very well.

30th.—Slight diarrhœa; otherwise well.

November 1st, three o'clock P.M.—Until this morning the child has been going on remarkably well, scarcely having suffered any thing from the repeated operations, which certainly always relieved it. About five o'clock this morning, the mother says, the child was suddenly seized with a violent attack of fever, hot skin, thirst, very restless, frequently screaming as if in pain, and the head very hot. These symptoms were followed, in the course of a few hours, by a very copious perspiration. I removed the bandage, and found the surface of the scalp red and inflamed, the head shining and tense; and the blood-vessels of the integuments, as also those on the eyes and nose, very turgid. The countenance expresses great anxiety; he screams often, and is very restless. Three evacuations yesterday, but none during the night or to-day; urine scanty; and pulse 120, and feeble. —To apply cold to the head constantly; and calomel, with saline aperients, to be taken internally.

Ten o'clock P.M.—Less heat on the head; has been purged; the pulse 135; less moaning.

2d.—Passed a tolerably good night; fever less; has had two evacuations, and has passed more urine; skin cool. Redness on the scalp much subsided, and the head is less hot and tense; countenance more composed. The tongue is white; and, although the pulse is 160, still the child seems much better. The circumference is twenty-two inches and a quarter.

3d.—Pulse 140. The erysipelas is subsiding, and the child is better.

4th.—Better.

5th, two o'clock P.M.—The child is now nearly in the same state of health as previous to the attack of erysipelas. The circumference is twenty-two inches and a quarter. I introduced the trocar on the right side; and, in rather less than five minutes, I drew off three ounces and a half of fluid. He bore this operation very well, and appeared to be much relieved by the evacuation of the fluid.—The application of cold to be continued.

Night.—Very comfortable.

The 6th, 7th, 8th, 9th, and 10th, were passed in good health, the child appearing free from complaint.

11th.—For the last twenty hours, the breathing has been laborious. The circumference of the head is twenty-three inches and a half. Introduced the trocar on the left side, and three ounces of fluid were drawn off in four minutes: the fluid escaped in a stream. I will here remark, that the fluid, at each subsequent operation, was clearer than at the previous one.

Half-past five.—The relief obtained is not so great as from former operations.

12th.—Very comfortable.

14th.—Removed to his own home, No. 5, Margaret-street, Bagnage Wells-road, Clerkenwell.

19th.—The scalp is inflamed around the two last punctures, and there is oozing from the one last made. The external veins are very turgid; the parietal and frontal bones are closer to each other; the fluctuation is less distinct. The circumference is twenty-three inches. Pulse 120. Respiration is to-day more hurried and shorter than of late. The bowels act sufficiently without medicines, and the parents consider the child much better.—The puncture to be deferred; a moderate firm bandage is applied, and cold application to be continued.

23d.—I learn that, a very short time after I made my last visit, about four ounces of fluid oozed out from the last-made opening, and that the respiration shortly became natural. The circumference is twenty-three inches and a half. Pulse 140; bowels natural; extremities warm; the countenance is improved, and the child is more intelligent, and sleeps much less. Fluctuation between the bones is less distinct, and the head consequently feels firmer: therefore, although the head is not diminished in the circumference, I have determined to postpone further measures for the present, to apply a bandage to the head as a support, and leave the case to nature.

26th.—Symptoms of pressure increased in a slight degree; otherwise the child is well.

30th.—He is drowsy and heavy, and other symptoms of compression are more manifest; but in other respects he is well. The circumference of the head is twenty-three inches and a half. From the anterior tip of one ear, the string being carried backwards to the same point of the other ear, is fifteen inches and a half. From the same point of the ear, carrying the string directly over the vertex to the same point of the other ear, is fifteen inches and a half. Made a puncture on the right side, and evacuated three ounces and seven drachms in eight minutes. The fluid is much clearer. The symptoms of compression subsided, and the bandages were applied as usual.

December 4th.—The physical functions natural, and the parents perceive a considerable increase of moral development, and are much gratified.

9th.—Symptoms of compression returning: the circumference of the head is twenty-three inches and a half. Punctured on the left side, and drew off three ounces and a half of fluid, in the course of four minutes and a half: the colour of the fluid paler. The health natural.

14th.—In every respect well.

20th.—General circumference of the head is twenty-three inches and a quarter. Introduced the trocar on the left side, and, in seven minutes and a half, four ounces of fluid were collected: it passed out almost *guttatim*. Health as before.—Applied the bandages as formerly.

27th.—He has had, within the night, frequent convulsions, and I find several teeth are forcing their way through the gums. He is wasted; but his bowels, pulse, and respiration, are little disturbed. The scalp is loose.—Ordered the gums to be scarified, and aperients to be taken.

28th.—Convulsions frequent and severe during the night, and in the interval he falls into a comatose state. The gums, through neglect, have not been lanced; but, upon lancing them, several teeth appeared, and the child became tranquil. General circumference is twenty-two inches and a half, and the scalp continues loose; but there is evident fluctuation. Punctured on the right side, and drew off, *guttatim*, two ounces in five minutes; but which was accomplished chiefly by pressing the sides of the cranium together.—Bandages as before.

30th.—Continues to get worse. Head as before.

January 5th.—He is much wasted. Frequent, violent, and severe convulsions, and after each he is quite exhausted. His pulse is small, and very quick; breathing hurried; bowels are now confined, and his evacuations are very offensive. He refuses his food. Upon removing the bandages to examine the head, a gurgling noise was heard; the scalp instantly became loose and flattened, and the bones overlapped one another, but the circumference was twenty-two inches.—Re-applied the firm and uniform bandage, and removed portions of the gums from several teeth. Cordials, and mercurial purgatives, were directed.

6th.—Convulsions continue, and the child is fast sinking.

7th.—He died.

*Sectio Cadaveris, fourteen Hours after Death.*

*The external appearances of the head.*—The scalp over the anterior fontanel was flat and sunk inwards, rendering the edges of the bone prominent. The circumference of the head was twenty-two inches. The coats of the left eye were ruptured.

*Internal appearances.*—The dura mater was much thickened, and it adhered very firmly to the inner surface of the cranium. The pia mater distinctly inflamed. The surface of the brain smooth, exhibiting no appearance of convolutions; and, on cutting into its substance, it was found to be only about one-sixth of an inch in thickness: nevertheless, it was composed both of cortical and medullary matter. There was no vestige of corpus callosum, corpus striatum, thalamus nervi optici, fornix, septum lucidum: in short, the various parts usually found in the cerebrum were entirely absorbed, and the space filled up by one large bag, which was lined by an adventitious white membrane, exceedingly firm, and of about the thickness of ordinary writing paper. The bag was divided into cells by broad, flat, membranous bands, taking the course of the lobes, and contained about thirty-five ounces of fluid. These bands appeared to be plexus choroides, or prolongations of the pia mater. By minutely inspecting the adventitious membrane, the small apertures made by the trocar at the different operations were visible; but similar ones could not be found in the small remaining substance of the brain, or in the dura mater.

The cerebellum was firmer than usual; as also the optic nerves at the fore-part of the sella turcica; but no other appearance existed worthy of notice.

*Hanover-street, Hanover-square; Nov.* 4, 1824.

